# The double-edge sword of CRISPR application for *in vivo* studies

**DOI:** 10.18632/oncotarget.28459

**Published:** 2023-11-27

**Authors:** Martin K. Thomsen

**Keywords:** CRISPR, *in vivo*, mouse models, cancer, Adeno-associated virus

A decade ago, the hallmark paper by Platt et al. was published on the *in vivo* application of Clustered Regularly Interspaced Short Palindromic Repeats (CRISPR) to generate cancer in different organs of mice. This paper outlines the advancement of delivering sgRNA’s to the target tissue to create loss or gain of function mutations without the need for timely intercrossing of genetic mouse strains. Furthermore, the study showed that multiplexing was possible, thereby enabling the method to target multiple sites simultaneously [1]. It was foreseen that this technology would change the way mouse models of cancer were generated, but even after 10 years, only few studies have relied on this methodology [2].

The double-edged sword of *in vivo* application of CRISPR is the imperfection of mutations generated in the target sequence. As CRISPR introduces mutations, they do not always occur, resulting in cells being present without the desired mutation. This is further complicated by the different types of indels, which can result in a functional protein with only changes in a few amino acids, without the introduction of a premature stop codon. This introduces clone-to-clone variation and results in tumors with a different mutation profile [3, 4]. However, this is also an advantage of CRISPR for generating *in vivo* cancer models as natural selection will occur, resulting in a cancer Darwinian evolution. Essential mutations will be present in the tumors, and negative selection can occur, keeping genes required for tumor progression intact. As an example, in prostate cancer, loss of Pten is always detected, underscoring the gatekeeper function of this gene [3]. We have also observed negative selection for loss of Foxa1 in the induction of prostate cancer, with a low mutation rate and reflected in an indolent phenotype when Foxa1 was found mutated [5]. The analysis of CRISPR-induced tumors provides unique insights into tumor biology, but due to the heterogeneity generated by CRISPR, it is necessary to genotype each tumor for the CRISPR-induced mutations [2].

**Figure 1:**
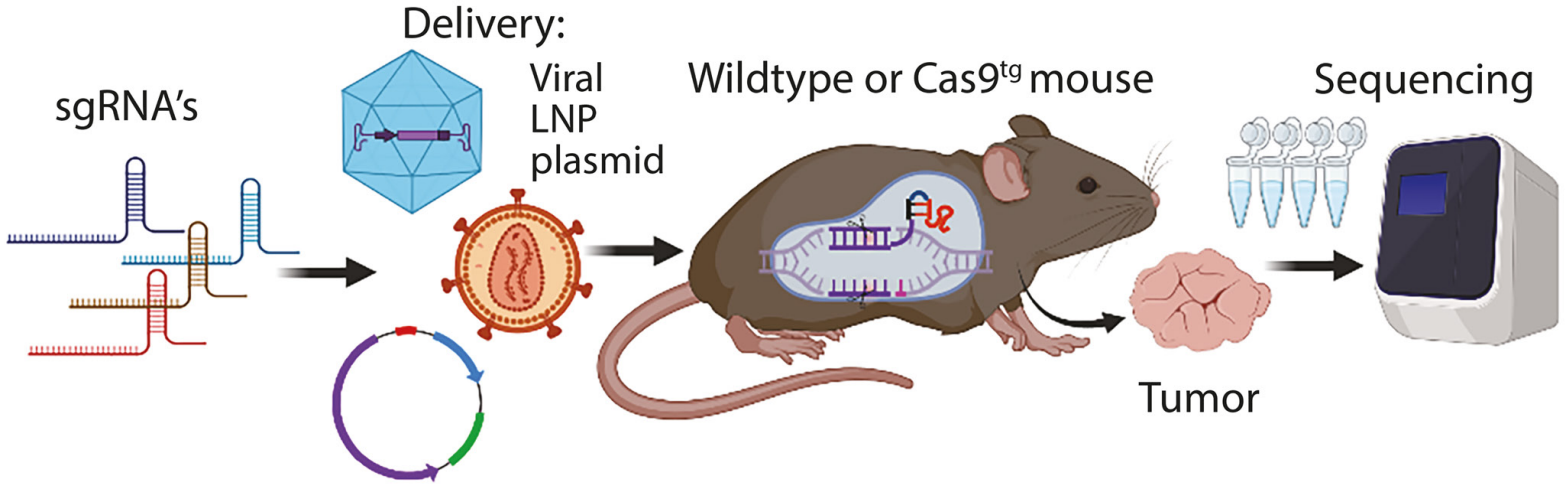
Workflow illustration. Unique single guide RNAs (sgRNAs) are delivered to the mouse by either viral particles, plasmids, or by lipid nanoparticles (LNPs). This can be combined with Cas9 protein and delivered to wild-type mice, or alternatively, a transgenic mouse for Cas9 can be used. Individual tumors must undergo sequencing at the target site for the sgRNAs to validate mutations introduced by CRISPR.

The Achilles’ heel of CRISPR application is the delivery of sgRNA/Cas9 to the desired tissues. Many approaches are used and vary between target tissues (Figure 1). Adeno-associated virus (AAV) is the most used method for delivery but has low carrier capacity. Other viral vectors such as lentivirus and adenovirus are used, but they come with disadvantages such as genomic integration and immunological activation [6, 7]. Lately, an effort has been made for the use of lipid nanoparticles to deliver sgRNA in complex with Cas9 or RNA coding for sgRNA and Cas9 [8]. Overall, generating particles for CRISPR delivery takes time and is a methodology burden with multiple pitfalls, which is hindering the use of CRISPR to generate *in vivo* cancer models. However, once the methodology is established, the generation of new particles targeting new genes is rapid and outcompetes classical mouse genetics by intercrossing different strains [9]. Researchers can include new sgRNA’s to their panel and apply them *in vivo* in a few weeks, progressing rapidly to address the question of interest at a low cost.

The application of CRISPR for *in vivo* studies has also shown new aspects of research into cancer. By using CRISPR, it has been possible to conduct *in vivo* screening for tumor suppressor genes in both hepatology and neurological cancer. Plasmid and AAV libraries have been successfully applied in combination with bioinformatic tools to reveal positive genetic interactions in cancer progression [10–12]. This type of experiment would never be feasible with classical mouse models, and future work will likely see these screens expand to CRISPR activation or inhibition screens. However, *in vivo* screens also have their limitations, as the number of targets that can be included is limited to ensure that the whole library is represented. The AAV library generated by Roland Rad group contains approximately 250 tumor suppressor genes, which ensure coverage and reproducibility of each target gene [10, 11].

CRISPR is now widely used for *in vitro* studies, and it will surely gain further use for *in vivo* studies. As the technique for *in vivo* delivery rapidly expands and becomes more accessible, one of the bottlenecks will disappear. The advantages of CRISPR for *in vivo* applications are numerous, including the rapid and cost-efficient generation of new genetic models of various cancers. As CRISPR can also be applied for *in vivo* screening, it offers new insights into tumor progression and resistance. Finally, genotyping of tumors is also rapidly developing, with decreased costs for Sanger and whole-genome sequencing, which will improve downstream analysis. Altogether, the *in vivo* application of CRISPR will become more common, even though the technique has challenges, it will only become more feasible in the future, allowing more researchers to apply this technology.
